# Epidemiological, clinical, and prognostic analysis of oral squamous cell carcinoma diagnosed and treated in a single hospital in Galicia (Spain): a retrospective study with 5-year follow-up

**DOI:** 10.4317/medoral.26047

**Published:** 2023-06-18

**Authors:** Iñaut Amezaga-Fernandez, José Manuel Aguirre-Urizar, José Manuel Suárez-Peñaranda, Cintia Chamorro-Petronacci, Irene Lafuente-Ibáñez de Mendoza, Xabier Marichalar-Mendia, Andrés Blanco-Carrión, José Antúnez-López, Abel García-García

**Affiliations:** 1 Department of Stomatology II. University of the Basque Country (UPV/EHU), Leioa, Spain; 2 Department or Forensic Sciences and Pathology. Faculty of Medicine and Dentistry. University of Santiago de Compostela. Santiago de Compostela, Spain. Department of Pathology. Clinical Hospital. Santiago de Compostela, Spain; 3 Instituto de Investigación Sanitaria de Santiago (IDIS). Santiago de Compostela, Spain; 4 Department of Nursery I, University of the Basque Country (UPV/EHU). Leioa, Spain; 5 Faculty of Medicine and Dentistry, University of Santiago de Compostela. Santiago de Compostela, Spain; 6 Maxillofacial Surgery Service, Complejo Hospitalario Universitario de Santiago. Santiago de Compostela, Spain

## Abstract

**Background:**

Oral cancer is a common neoplasm worldwide, mostly corresponding to squamous cell carcinoma (OSCC). Unfortunately, its overall prognosis remains poor, with no improvement in recent decades. In this study, we have analysed the epidemiological, clinical, and prognostic characteristics of OSCC on patients of a specific Spanish region (Galicia), in order to improve its prognosis and apply effective preventive and early diagnosis measures.

**Material and Methods:**

We retrospectively analysed 243 cases of OSCC, diagnosed and treated in a single hospital centre in Galicia between 2010 and 2015 (minimum of 5 years of evolution). Overall and specific survival were calculated (Kaplan-Meier) and associated variables were identified (log rank test and Cox regression).

**Results:**

The mean age of the patients was 67 years, with the majority being male (69.5%), smokers (45.9%) and alcohol consumers (58.6%), who lived in non-urban areas (79.4%). Cases diagnosed at advanced stages entailed the 48.1% of the sample, and 38.7% of cases relapsed. The 5-year overall and disease-specific survival rates were 39.9% and 46.1%, respectively. Patients who consumed tobacco and alcohol had a worse prognosis. OSCC cases referred to hospital by specialist dentists had a better prognosis, as those who were previously diagnosed with an oral potentially malignant oral disorder (OPMD) or received dental care during OSCC treatment.

**Conclusions:**

In view of these findings, we conclude that OSCC in Galicia (Spain) still has a very poor overall prognosis, which is mainly related to the advanced age of the patients and the late diagnosis. Our study highlights the better survival of OSCC in relation to the referring health professional, the presence of a previous OPMD and the dental care after diagnosis. This demonstrates the importance of dentistry as a health profession involved in the early diagnosis and multidisciplinary management of this malignant neoplasm.

** Key words:**Oral squamous cell carcinoma, epidemiology, clinical study, diagnosis, prognosis, survival, cancer, oral.

## Introduction

According to the Global Cancer Observatory, 377,173 new cases of oral cancer were diagnosed worldwide in 2020, placing oral cancer as the 16th most common cancer (1). In Spain, incidence of oral cancer is high, with 10.3 cases per 100.000 citizens. Together with the oropharynx, it is positioned among the 10 most common malignancies (1).

Squamous cell carcinoma (SCC) is the most frequent type of oral cancer, accounting for more than 90% of cases (2). It is mainly diagnosed in men aged between 50-70 years, although an increase in incidence in women has been observed in recent decades (3). The most important risk factors for this neoplasm are tobacco and alcohol, to which 64% of all OSCC are ascribed (4,5). Other aetiological factors are human papillomavirus infection or actinic radiation (4).

Oral SCC (OSCC) usually presents as an ulcerated tumour appearing at any location, but the most affected areas are the lateral border of the tongue, floor of mouth and gingiva (6). On some occasions, malignant lesions are preceded by oral potentially malignant disorders (OPMD), such as leukoplakia, oral lichenoid disease, etc (7). Despite this, half of OSCC patients are diagnosed in late clinical stages (6). Consequently, mortality of OSCC is high, with 5-year survival rates below 50% (8). Tumour staging (TNM) is the most important predictor of prognosis, specially cervical lymph node involvement (6). Other factors affecting the outcome of OSCC include sex, anatomic location and some histopathologic characteristics (6). Treatment is based on the surgical excision of the tumour and cervical dissection. Adjuvant therapies such as radiotherapy and chemotherapy are implemented in advanced stages.

Galicia, an autonomous region in the north-western end of Spain, has demographic particularities in comparison to other areas. It has one of the oldest citizenries, with 26% of the inhabitants being older than 65 years, and one of the regions with highest general cancer incidence (732 new cases per 100.000 citizens) (9). Moreover, the percentage of tobacco consuming population is lower than in the rest of Spain; however, it is one of the regions with highest daily alcohol consumption. These facts suggest that oral cancer in this region could have particular characteristics regarding epidemiology, clinicopathological patient profile and even survival. Nevertheless, there are no reliable data sources for oral cancer in this territory.

Against this background, we decided to plan a retrospective study of OSCC in a cohort of patients diagnosed and treated in a single hospital, with the aim of identifying its main epidemiological, clinical, and prognostic characteristics. This would facilitate the outlining of a protocol for prevention and early diagnosis in order to improve the prognosis of this malignancy.

## Material and Methods

We designed an observational retrospective study, including all patients diagnosed and treated for OSCC at the University Hospital Complex of Santiago de Compostela (CHUS), between the years 2010 and 2015. All patients were diagnosed histopathologically by the Department of Pathology of CHUS. A minimum of 5 years of follow-up after diagnosis was required for inclusion.

A specific protocol was designed in which the main epidemiological, clinical, diagnostic, therapeutic and evolutionary data of each case were included. The principal variables collected from the medical records were age, sex, residential setting, origin of referral, smoking (current or past), alcohol consumption (current or past), tumour location, clinical appearance, presence and type of previous OPMD, TNM staging, treatment modality, degree of differentiation, recurrence, development of a second primary tumour (oral cavity or other locations), death and cause of death.

Residential settings were divided in three groups: cities (densely populated), towns and suburbs (intermediate density) and rural areas (thinly populated). Smoking and drinking status were assessed as follows: current, past (less than 10 years from cessation), non-consumers (including past consumers over 10 years from cessation). TNM staging and histopathological analysis were performed according to the guidelines of the 7th edition of the AJCC Cancer Staging Manual (10). The date of diagnosis and date of death or last follow-up were also recorded to calculate survival.

For the statistical analysis, we used IBM SPSS Statistics software (version 28.0). Initially, we carried out a descriptive analysis of the group, calculating frequencies and percentages for qualitative variables and the mean and Standard Deviation (SD) for quantitative variables. For survival analysis, Kaplan-Meier curves were constructed for overall 5-year survival (OS) and disease-specific survival (DSS). To identify the factors related to OS and DSS, survival curves were compared using log rank test. Also, Cox proportional hazards regression model was constructed for OS and DSS. Statistical significance was established at *p* ≤ 0.05 values.

## Results

- Descriptive analysis

A total of 243 patients were included in the study. The main epidemiological and clinicopathological characteristics are listed in Table 1.

**Table 1:**
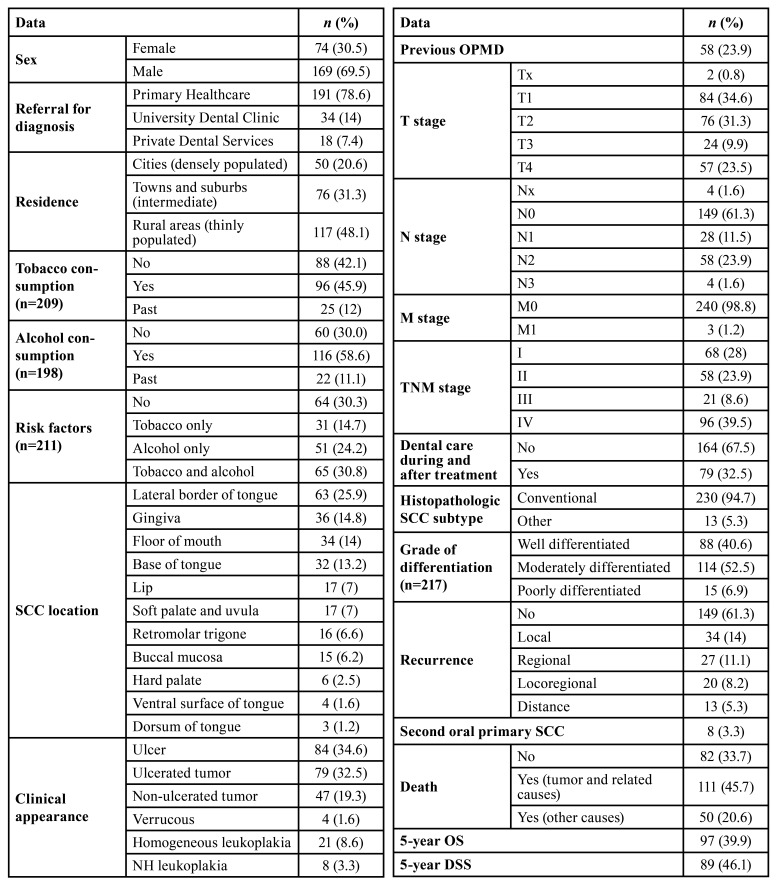
Epidemiological, clinicopathological and prognostic features.

Mean age at the time of diagnosis was 67.02±12.39 years (range 20-98), with 82 patients (33.7%) being younger than 60 years, and just one patient (0.4%) younger than 40 years. The study group was composed of 74 women (30.5%) and 170 men (69.5%).

Most patients lived in rural or suburban areas (79.4%), whereas 20.6% resided in cities, making OSCC 4 times more common in individuals residing in non-densely populated areas.

Patients were referred for diagnosis mainly from the Primary Healthcare of the Galician Public Health System (SERGAS) (78.6%).

Tobacco and alcohol consumption was reported by 96 (45.9%) and 116 (58.6%) patients, respectively. Those referring both habits were 65 (30.8%). Past smokers (<10 years) were 12% and former drinkers 11%. In 34 (14%) and 45 (18.5%) cases there was no data on tobacco and alcohol consumption, respectively.

The most frequent location of the primary OSCC was the tongue (41.9%), specifically the lateral border (25.9%), followed by the gingiva (14.8%) and floor of the mouth (14%).

The main clinical presentations were the ulcer (34.6%) and the ulcerated tumour (32.5%). Considering previous oral lesions, 58 patients (23.9%) presented an OPMD, mostly oral leukoplakia (61.4%).

At the time of diagnosis, the majority of the tumours were T1 or T2 (34.6% and 31.3%, respectively). Nodal involvement was present in 90 patients (38.9%); in addition, 1.2% presented distant metastases (M1). Regarding the overall stage, 28% were classified as stage I, 23.9% stage II, 8.6% stage III and 39.5% stage IV.

Histopathological analysis revealed that most tumours (94.7%) were conventional SCCs, principally with well to moderate differentiation (93.1%).

Regarding 5-year outcome, 94 patients (38.7%) suffered a recurrence, and 3.3% developed a second oral primary SCC. Five-year OS and DSS rates were 39.9% and 46.1%, respectively. Death from OSCC or related causes occurred in 45.7% while the rest (20.6%) died of unrelated causes. Mean overall and disease-specific survival times were 57.37 (CI=51.08-63.66) and 65.14 (CI=57.72-72.57) months, respectively.

- Factors related to survival

The main variables associated with survival are shown in Table 2.

Using the log rank test, we observed that the following features were related to OS: origin of referral, T and N stage, overall stage, development of recurrence and dental care during oncologic treatment (Table 2). Besides, women had a higher OS than men, although the difference was not statistically significant (*p*=0.057).

**Table 2:**
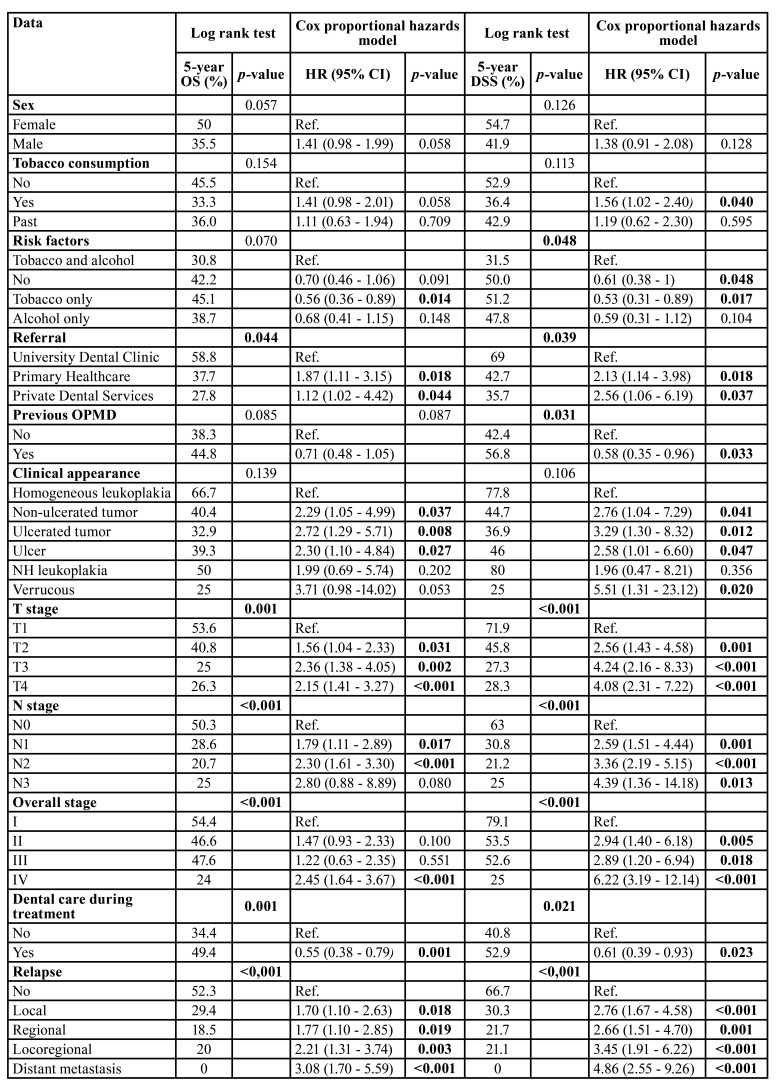
Log rank test and Cox proportional hazards model for variables associated to 5-year OS and DSS.

After performing regression analysis regarding OS, a better survival was found in cases referred to the hospital from the Dental Clinic of the University of Santiago de Compostela (USC) compared to those referred from the primary healthcare (HR=1.87; CI=1.11-3.15; *p*=0.018) or private dental clinics (HR=1.12; CI=1.02-4.42; *p*=0.044). Also, patients who received dental care during OSCC treatment had a better OS (HR=0.55; CI=0.38-0.79; *p*=0.001). Regarding toxic habits, tobacco and alcohol consumption implied a worse OS than tobacco alone (HR=0.56; CI=0.36-0.89; *p*=0.014).

In relation to the clinical aspect at the time of diagnosis, cases in which OSCC appeared as a homogeneous leukoplakia had a better prognosis than as an ulcer (HR=2.30; CI=1.10-4.84; *p*=0.027), a tumour (HR=2.72; CI=1.29-5.71; *p*=0.008) or an ulcerated tumour (HR=2.29; CI=1.05-4.99; *p*=0.037). In addition, cases diagnosed at earlier stages, including tumour size (T), positive lymph nodes (N) and overall stage had a better overall prognosis, as well as the absence of recurrence.

We recognised similar associations for DSS with log rank (Table 2). However, we identified data related to worse survival, such as tobacco and/or alcohol consumption (*p*=0.048). In contrast, the presence of an OPMD was associated with better DSS (*p*=0.031).

In the regression model, patients with OSCC who smoked had a worse DSS than non-smokers (HR=1.56; CI=1.02-2.40; *p*=0.040). We observed that patients who smoked and drank had a worse DSS than those who did not (HR=0.61; CI=0.38-1.00; *p*=0.048) and those who only smoked (HR=0.53; CI=0.31-0.89; *p*=0.017). The remaining variables related to DSS were the same as for OS.

We did not recognise any association in relation to survival (both OS and DSS) with patient age, residential setting, alcohol consumption alone or histopathological differentiation.

## Discussion

This study analyses the largest cohort of patients with oral squamous cell carcinoma in Galicia, a Spanish region with particular socio-cultural aspects and a high overall incidence of cancer (9).

Our study shows that OSCC in Galicia affects patients with a slightly older mean age (67 years) than that observed in other countries (6,11). However, it is similar to that of other Spanish studies (12,13) and to those conducted in Galicia (14,15). It is noteworthy that only one patient younger than 40 years appeared in our cohort, which contrasts with other studies (11,12). We believe that these results may be related to the higher life expectancy in this region, which exceeds 80 years (9); as well as to the fact that many diagnoses of OSCC have been late (48.1% in stages III and IV), allowing oral carcinogens intensely associated with tobacco and/or alcohol consumption to remain active for a longer period of time.

We have not been able to recognise significant differences with respect to sex in our study. OSCC in Galicia is more common in men, as observed by other authors (12,14). However, we found that women with OSCC showed a better survival, and despite not being significant in our study, other authors have recognised this difference in prognosis (11,14,16). This observations could be related to a lower consumption of tobacco and/or alcohol, as well as the earlier diagnosis of OSCC in women, as suggested in other studies conducted in Galicia (17,18).

In Galicia, a large majority of patients with OSCC are regular consumers of tobacco and/or alcohol, which remains the most important known oral carcinogenic binomial, and whose consumption numbers have been similar to those in other studies (6,12). However, the high frequency of alcohol consumption in our cohort is striking, a fact found in this region in recent epidemiological reports (9). Similar to what has been observed by other authors (2,19), we have found that tobacco use is associated with poorer survival. Also, we have recognised that the carcinogenic effect of tobacco is reversible, as our ex-smoking patients with OSCC had a DSS similar to that of non-smoking patients (2). A very important result that we obtained was the poor prognosis associated with the joint consumption of tobacco and alcohol, which would once again highlight the carcinogenic potentiation of this binomial in oral cancer (20).

In our work we have been able to verify that most patients with OSCC in Galicia reside in non-densely populated areas (rural and suburban), which contrasts with studies on head and neck cancer in Spain, reported to be more common in densely populated (urban) areas (21). We believe that this could be due to the demographic particularities of Galicia, a region with a large percentage of rural population, with greater tobacco and/or alcohol consumption and singular socio-cultural and health aspects (9). However, we have not found any relationship between residential setting and survival, in line with the contradictory results obtained in this aspect (22,23).

As in previous studies (18,24), the type of healthcare professional who made the initial diagnosis of OSCC has proven to be very relevant for prognosis. Cases referred to the hospital from the University of Santiago de Compostela (USC) Dental Clinic by specialist dentists had a significantly higher survival rate. We consider this result to be a wake-up call for health professionals, whether dentists or not, private or public, to be properly trained in the early diagnosis of this cancer. Once again, our study also demonstrates that a better prognosis in OSCC is significantly related to a small tumour size, i.e., early diagnosis of this cancer, as do some studies (6,12,25).

As expected, the most frequent location of OSCC was the tongue, preferentially the lateral border, followed by the gingiva and the floor of the mouth, as in other studies conducted in Spain (12,13,17).

Almost a quarter of our patients had been diagnosed with an OPMD prior to the OSCC, which reinforces the importance of a good early diagnosis of these oral lesions and their proper control and management (26). Oral leukoplakia was the most commonly diagnosed OPMD, in agreement with other studies carried out in Spain (12,17,27). These associated cases had a higher DSS, which could be explained by early cancer detection and therapy, achieved during the disorder follow-up, as previously reported (27).

The most common clinical appearance of OSCC in our study was the ulcer, with or without tumour, similar to others (11,12,27). A significant number of our cases presented clinically as leukoplakia (11.9%), both homogeneous and non-homogeneous. Surprisingly, these cases had a higher 5-year survival, which in our opinion justifies the importance of always making a good clinicopathological diagnosis and control of these OPMDs (28).

Undoubtedly, early diagnosis is one of the main objectives in the Fight against cancer, but in the case of OSCC it is crucial, although this is far from being achieved (12). We recognised that half of the patients in our study were diagnosed at an advanced clinical stage, which means that the overall prognosis of this cancer remains very poor in Galicia. The percentage of cases diagnosed at early stages was similar to other studies in this area previously published (14,17). These data point to the need to implement specific preventive protocols with the widest possible social diffusion to prevent and early diagnose this cancer.

The majority of our tumours were histopathologically differentiated (93.1%), with no significant correlation with survival, which questions again the prognostic value of this classification in OSCC (29).

An interesting result of our study was the better prognosis of patients who received oral care after diagnosis and during oncologic treatment of OSCC. This finding has recently been corroborated in a meta-analysis by Haynes *et al*. (30); highlighting the importance of oral and dental care in patients with oral cancer, as well as the relevance of dental professionals within the multidisciplinary team involved in its management.

OSCC in Galicia continues to be a malignant neoplastic disease with a poor prognosis, with a low overall survival at 5 years (39.9%); lower than in other regions of Spain and Europe (8,12,13). This adverse prognosis could be partly explained by the high percentage of elderly patients in our study, which would lead to higher mortality, as has also been noted (19). In addition, our study has a high number of patients who were diagnosed at advanced stages, which is accompanied by a high rate of local and regional recurrences and reduced effectiveness of therapies (25).

To our knowledge, this study is the largest conducted on OSCC in Galicia (Spain) with the aim of understanding its epidemiological, clinical and prognostic profile. We believe that the fact that all patients were treated in the same hospital favours the homogeneity of the sample, in terms of their origin and the therapeutic protocols. This retrospective study has some inherent limitations, such as the loss of some data, which could not be recovered. Survival at 5 years post-therapeutic evolution may also be a limitation; nonetheless, we wanted data homogeneity, which is considered a key in the general assessment of cancer. Another current limitation is that the staging of patients was based on the 7th edition of the AJCC Cancer Staging Manual (10), and we were unable to update it to the 8th edition due to the absence of some fundamental data that did not appear in the previous edition.

In summary, this study has allowed us to characterise the epidemiological, clinical and prognostic profile of OSCC in Galicia, highlighting particular aspects related to the demographic and socio-cultural singularities of this region. OSCC in Galicia affects more elderly people, mainly men, living in non-urban areas, and shows a close relationship with tobacco and/or alcohol consumption. The overall prognosis of OSCC in Galicia is poor, worse than in other regions, which could be related to the high percentage of late diagnosis and the advanced age of the patients. The importance of the dentist in the diagnosis and management of OSCC should be emphasised, considering that patients with previous OPMDs and those receiving dental care during cancer treatment showed an improved prognosis. The association between good survival, the origin of hospital referral and the early diagnosis and treatment, highlights the need to schedule continuous training for health professionals (dentists and doctors) on this aggressive malignant pathology, which will allow us to obtain better results in the future.
